# Supplementary material for: Heat adaptation of phage T7 under an extended genetic code
Source: Virus Evol. 2021 Dec 1;7(2):veab100. doi: 10.1093/ve/veab100 (PMC8923235; doi:10.1093/ve/veab100)
Supplement: veab100_Supp [file veab100_supp.zip › Sequencing_Analysis.zip!/Sequencing_Analysis/S3/evidence/SNP_8.html]

BRESEQ :: Evidence


| Predicted mutation | | | | | | |
| --- | --- | --- | --- | --- | --- | --- |
| evidence | seq id | position | mutation | annotation | gene | description |
| RA | NC\_001604.1 | 21,734 | A→G | intergenic (–/–) | – / – | –/– |

| Read alignment evidence... | | | | | | | | | | | |
| --- | --- | --- | --- | --- | --- | --- | --- | --- | --- | --- | --- |
|  | seq id | position | | ref | new | freq | score (cons/poly) | reads | annotation | genes | product |
| \* | NC\_001604.1 | 21,734 | 0 | A | G | 98.1% | 4713.7 / 23.1 | 1594 | intergenic (–/–) | *–/–* | –/– |
| Reads supporting (aligned to +/- strand):  **ref** base A (17/13);  **new** base G (719/840);  **total** (739/855) | | | | | | | | | | | |
| Fisher's exact test for biased strand distribution *p*-value = 2.72e-01 | | | | | | | | | | | |
| Kolmogorov-Smirnov test that lower quality scores support variant *p*-value = 8.18e-01 | | | | | | | | | | | |

Only 100 of 1594 total aligned reads displayed.  

`CTCTGGTACGAGTGCTCTTGAAGCAACTACAAGCCACGCAACAGATTCCTGAGTTACCTAAGGAAGCCGTAGAGCCAACCATTAGTACAGGTCTGGAAGCAATTGGTCGAGGACAAGACCTTGATAAGCTGGAGCGGTGTGTCACTGCGTGGGCTGCACTGGCACCTATGCGGGACGACCCTGATATTAACCTTGCGATGATTAAGTTACGTATTGCCAACGCTATCGGTATTGACACTTCTGGTATTCTACTCACCGAAGAACAGAAGCAACAGAAGATGGCCCAACAGTCTATGCAAATGGGTATGGATAATGGTGCTGCTGCGCTGGCTCAAGGTATGGCTGCACAAGCTACAGCTTCACCTGAGGCTATGGCTGCTGCCGCTGATTCCGTAGGTTTACAGCCGGGAATTTAATACGACTCACTATAGGGAGACCTCATCTTTGAAATGAGCGATGACAAGAGGTTGGAGTCCTCGGTCTTCCTGTAGTTCAACTTTAAGGAGACAATAATAATGGCTGAATCTAATGCAGACGTATATGCATCTTTTGGCGTGAACTCCGCTGTGA‑TGTCTGGTGGTTCCGTTGAGGAACAT  >  NC_001604.1/21435‑22030`  

                                                                                                                                                                                                                                                                                                           `|`                                                                                                                                                                                                                                                                                                           
    `ggTACGAGTGCTCTTGAAGCAACTACAAGCCACGCAACAGATTCCTGAGTTACCTAAGGAAGCCGTAGAGCCAACCATTAGTACAGGTCTGGAAGCAATTGGTCGAGGACAAGACCTTGATAAGCTGGAGCGGTGTGTCACTGCGTGGGCTGCACTGGCACCTATGCGGGACGACCCTGATATTAACCTTGCGATGATTAAGTTACGTATTGCCAACGCTATCGGTATTGACACTTCTGGTATTCTACTCACCGAAGAACAGAAGCAACAGAAGATGGCCCAACAGTCTATGCAAGTGGGt                                                                                                                                                                                                                                                                                                      <  1:242539/301‑1 (MQ=255)`  
     `gTACGAGTGCTCTTGAAGCAACTACAAGCCACGCAACAGATTCCTGAGTTACCTAAGGAAGCCGTAGAGCCAACCATTAGTACAGGTCTGGAAGCAATTGGTCGAGGACAAGACCTTGATAAGCTGGAGCGGTGTGTCACTGCGTGGGCTGCACTGGCACCTATGCGGGACGACCCTGATATTAACCTTGCGATGATTAAGTTACGTATTGCCAACGCTATCGGTATTGACACTTCTGGTATTCTACTCACCGAAGAAAAGAAGCAACAGAAGATGGCCCAACAGTCTATGCAAGTGGGTa                                                                                                                                                                                                                                                                                                     >  1:130585/1‑301 (MQ=255)`  
         `gggggCTCTTGAAGCAACTACAAGCCACGCAACAGATTCCTGAGTTACCTAAGGAAGCCGTAGAGCCAACCATTAGTACAGGTCTGGAAGCAATTGGTCGAGGACAAGACCTTGATAAGCTGGAGCGGTGTGTCACTGCGTGGGCTGCACTGGCACCTATGCGGGACGACCCTGATATTAGCCTTGCGATGATTAAGTTACGTATTGCCGACGATATCGGTATTGACACTTCTGGTATTCTACTCACCGAAGAACAGAAGCAACAGAAGATGGCCCAACAGTCTATGCAAGTGGGTATGGa                                                                                                                                                                                                                                                                                                 <  1:214964/297‑1 (MQ=255)`  
          `agtgattgttaaGCAACTACAAGCTACGCAACAGAGATCTGAGTTAGCTGAGGAAGAAGTATAGCCAACCATTAGTACAGGTCGGGAAGCAATTGGTCGAGGACAAGACCTTGATAAGCTGAAGCGGTGTGTCACTGCGTGTGCTGCACTGGCACCTATGCGGGAAGACCCTGATATTAACCTTGCGATGATTAAGTTACGTATTGCCAACGCTATCGGTATTGACACTTCTGGTATTCTGCTCACCGAAGAACAGAAGCAACAGAAGATGGCCCAACAGTCTATGCAAGTGGGTATGGAt                                                                                                                                                                                                                                                                                                <  1:341369/291‑1 (MQ=255)`  
          `aGTGCTCTTGACGCAACTACAAGCCACGCAACAGATTCCGGAGTTACCAAAGGAAGCCGTAGAGCCAACCATTAGGAAAGGTCTGGAAGCAATTGGTCGAGAACAAGACCTTGATAAGCTGGCGCGGTGTGTCACTGCGTGGGCTGCACTGGCACCTATGCGGGACGACCCTGTTATTAACCTTGCTATGATTAAGTTACGTATTGCCAACGCTATCGGTATTGACACTTCTGGTATTCTACTCACCGAAGAACAGAAGCAACAGAAGATGGCACAACAGTCTATGCAAGTGGGTATGGAt                                                                                                                                                                                                                                                                                                <  1:45658/301‑1 (MQ=255)`  
          `aGTGCTCTTGAAGAAACTACAGACCACGCAACAGATTCCTGAGTTACCAAAGGAAGCCGTAGAGCCAACCATTAGTACAGGTCTGGAAGCAATTGGTCGAGGACAAGACCTTGATATGCTGGAGCGGTGTGTCACTGCGTGGGCTGCACTGGCACCTATGCGGGACGACCCTGATATTAACCTTGCGATGATTAAGTTACGTATTGCCAACGCTATCGGTATTGACACTTCTGGTATTCTACTCACCGAAGAACAGAAGCAACAGAAGATGGCCCAACAGTCTATGCAAGTGGGTATGGAt                                                                                                                                                                                                                                                                                                <  1:147710/301‑1 (MQ=255)`  
               `tcttaaagcagatcaaaGCCACGCAACAGGTTTCTGAGTTGCCTAAGGAAGCCGTAGAGCCAACCATTAGTGCAGGTCTGGAAGCAATTGGTCGAGGACAAGACCTTGATAAGCTGGAGCGGTGTGTCACTGCGTGGGGTGCACTGGAACCTATGAGGGACAACCCTGGTATTAATCTTGCGATGATTAAGTTACGTATTGCCGACGCTATCGGTATTGACACTTCTGGTATTCTACTCACCGAAGAACAGAAGCAACAGAAGATGGCCCAACAGTCTATGCAAGTGGGTATGGATAATgg                                                                                                                                                                                                                                                                                           <  1:66953/286‑1 (MQ=255)`  
               `tctTGAAGCAACTACAAGCCACGCAACAGATTCCTGAGTTACCTAAGGAAGCCGTAGAGCCAACCATTAGTACAGGTCTGGAAGCAATTGGTCGAGGACAAGACCTTGATAAGCTGGAGCGGTGTGTCACTGCGTGGGCTGCACTGGCACCTATGCGGGACGACCCTGATATTAACCTTGCGATGATTAAGTTACGTATTGCCAACGCTATCGGTATTGACACTTCTGGTATTCTACTCACCGAAGAACAGAAGCAACAGAAGATGGCCCAACAGTCTATGCAAGTGGGTATGGATAATgg                                                                                                                                                                                                                                                                                           <  1:9575/301‑1 (MQ=255)`  
               `tctTGAAGCAACTACAAGCCACGCAACAGATTCCTGAGTTACCTAAGGAAGCCGTAGAGCCAACCATTAGTACAGGTCTGGAAGCAATTGGTCGAGGACAAGACCTTGATAAGCTGGAGCGGTGTGTCACTGCGTGGGCTGCACTGGCACCTATGCGGGACGACCCTGATATTAACCTTGCGATGATTAAGTTACGTATTGCCAACGCTATCGGTATTGACACTTCTGGTATTCTACTCACCGAAGAACAGAAGCAACAGAAGATGGCCCAACAGTCTATGCAAGTGGGTATGGATAATgg                                                                                                                                                                                                                                                                                           <  1:310585/301‑1 (MQ=255)`  
               `aaagggacagaCTAAAAGCCACGAAACAGATTCCTGAGTTACCTAAGGAAGCCGTAGAACCAACCATTAGTACAGGTCTGGAAGCAATTGGTCGAAGACAAGACCTTGATAAGCTGGAGCGGTGTGTCACTGAGTGGGCTGCACTGGCACCTATGCGGGACGACCCTGATATTAACCTTGCGATGATTAAGTTACGTATTGCCAACGCTATCGGTATTGACACTTCTGGTATTCTCCTCACCGAAGAACAGAAGCAACAGAAGATGGCCCAAAAGTCTATGCAAGTGGGTATGGATAATgg                                                                                                                                                                                                                                                                                           <  1:107928/291‑1 (MQ=255)`  
                                        `aCAGATTCCTGAGTTACCTAAGGAAGCCGTAGATCCAACCATTAGTACAGGTCTAGAAGCAATTGGTCGAGGACAAGACCTTGATAAGCTGGAGCGGTGTGTCACTGCGTGGGCTGCACTGGCACCTATGCGGGACGACCCTGATATTAACCTTGCGATGATTAAGTTACGTATTGCCAACGCTATCGGTATTGACACTTCTGGTATTCTACTCACCGAAGAACAGAAGCAACAGAAGATGGCCCAACAGTCTATGCAAGTGGGTATGGATAATGGTGCTGCTGCGCTGGCTCAAGGTATg                                                                                                                                                                                                                                                                  >  1:127812/1‑301 (MQ=255)`  
                                               `ccTGAGTTACCTAAGGAAGCCGTAGAGCCAACCATTAGTACAGGTCTGGAAGCAATTGGTCGAGGACAAGACCTTGATAAGCTGGAGCGGTGTGTCACTGCGTGGGCTGCACTGGCACCTATGCGGGACGACCCTGATATTAACCTTGCGATGATTAAGTTACGTATTGCCAACGCTATCGGTATTGACACTTCTGGTATTCTACTCACCGAAGAACAGAAGCAACAGAAGATGGCCCAACAGTCTATGCAAGTGGGTATGGATAATGGTGCTGCTGCGCTGGCTCAAGGTATGGCTGcac                                                                                                                                                                                                                                                           <  1:269980/301‑1 (MQ=255)`  
                                                  `gAGTTACCTAAGGAAGCCGTAGAGCCAACCATTAGTACAGGTATGGAAGCAATTGGTCGAGGACAAGACCTTGATAAGCTGGAGCGGTGTGTCACTGCGTGGGCTGCACTGGCACCTATGCGGGACGACCCTGATATTAACCTTGCGATGATTAAGTTACGTATTGCCAACGCTATCGGTATTGACACTTCTGGTATTCTACTCACCGAAGAACAGAAGCAACAGAAGATGGCCAAACAGTCTATGCAAGTGGGTATGGATAATGGTGCTGCTGCGCTGGCTctgtctcttatacacatct                                                                                                                                                                                                                                                        >  1:191533/1‑283 (MQ=255)`  
                                                  `gAGTTACCTAAGGAAGCCGTAGAGCCAACCATTAGCACAGGTCTGGAAGCAATTGGTCGAGGACAAGACCTTGATAAGCTGGAGCGGTGTGTCACTGCGTGGGCTGCACTGGCACCTATGCGGGACGACCCTGATATTAACCTTGCGATGATTAAGTTACGTATTGCCAACGCTATCGGTATTGACACTTCTGGTATTCTACTCACCGAAGAACAGAAGCAACAGAAGATGGCCCAACAGTCTATGCAAGTGGGTATGGATAATGGTGCTGCTGCGCTGGCTCAAGGTATGGCTGCACAAg                                                                                                                                                                                                                                                        >  1:224736/1‑301 (MQ=255)`  
                                                         `ccaaGGAACACTTAGAGCCAACCATTAGTACAGGTCTGGAAGCAATTGGTCTAGGACAAGACCTTGATCAGCTGGAGCGGTGTGTCACTGCGTGGGCTGCACTGGCACCTATGCGGGACGACCCTGATATTAACCTTGCGATGATTAAGTTACGTATTGCCAACGCTATCGGTATTGACACTTCTGGTATTCTACTCACCGAAGAACAGAAGCAACAGAAGATGGCCCAACAGTCTATGCAAGTGGGTATGGATAATGGTGCTGCTGCGCTGGCTCAAGGTATGGCTGCACAAGCTACAGc                                                                                                                                                                                                                                                 <  1:230551/299‑1 (MQ=255)`  
                                                           `agatgtgtataagagACAGCCATTAGTACAGGTCTGGAAGCAATTGGTCGAGGACAAGACCTTGATAAGCTGGAGCGGTGTGTCACTGCGTGGGCTGCACTGGCACCTATGCGGGACGACCCTGATAATAACCTTGCGATGATTAAGTTACGTATTGCCAACGCTATCGGTATTGACACTTCTGGTATTCTACTCACCGAAGAACAGAAGCAACAGAAGATGGCCCAACAGTCTATGCAAGTGGGTATGGATAATGGTGCTGCTGCGCTGGCTCAAGGTATGGCTGCACAAGCTACAGCtt                                                                                                                                                                                                                                               <  1:56238/290‑1 (MQ=255)`  
                                                           `aaGGAAGCCGTAGAGCCAACCATTAGTACAGGTCTGGAAGCAATTGGTCGAGGACAAGACCTTGATAAGCTGGAGCGGTGTGTCACTGCGTGGGCTGCACTGGCACCTATGCGGGACGACCCTGATATTAACCTTGCGATGATTAAGTTACGTATTGCCAACGCTATCGGTATTGACACTTCTGGTATTCTACTCACCGAAGAACAGAAGCAACAGAAGATGGCCCAACAGTCTATGCAAGTGGGTATGGATAATGGTGCTGCTGCGCTGGCTCAAGGTATGGCTGCACAAGCTACAGCtt                                                                                                                                                                                                                                               <  1:17768/301‑1 (MQ=255)`  
                                                                 `gCCGTAGAGCCAACCATTAGTACAGGTCTGGAAGCAATTGGTCGAGGACAAGACCTTGATAAGCTGGAGCGGTGTGTCACTGCGTGGGCTGCACTGGCACCTATGCGGGACGACCCTGATATTAACCTTGCGATGATTAAGTTACGTATTGCCAACGCTATCGGTATTGACACTTCTGGTATTCTACTCACCGAAGAACAGAAGCAACAGAAGATGGCCCAACAGTCTATGCAAGTGGGTATGGATAATGGTGCTGCTGCGCTGGCTCAAGGTATGGCTGCACAAGCTCAAGCTTCACCTg                                                                                                                                                                                                                                         >  1:180745/1‑301 (MQ=255)`  
                                                                  `ccGTAGAGCCAACCATTAGTACAGGTCTGGAAGCAATTGGTCGAGGACAAGACCTTGATAAGCTGGAGCGGTGTGTCACTGCGTGGGCTGCACTGGCACCTATGCGGGACGACCCTGATATTAACCTTGCGATGATTAAGTTACGTATTGCCAACGCTATCGGTATTGACACTTCTGGTATTCTACTCACCGAAGAACAGAAGCAACAGAAGATGGCCCAACAGTCTATGCAAGTGGGTATGGATAATGGTGCTGCTGCGCTGGCTCAAGGTATGGCTGCACAAGCtctgtctcttataca                                                                                                                                                                                                                                        >  1:227523/1‑287 (MQ=255)`  
                                                                              `ccATTAGTACAGGTCTGGAAGCAATTGGTCGAGGACAAGACCTTGATAAGCTGGAGCGGTGTGTCACTGCGTGGGCTGCACTGGCACCTATGCGGGACGACCCTGATATTAACCTTGCGATGATTAAGTTACGTATTGCCAACGCTATCGGTATTGACACTTCTGGTATTCTACTCACCGAAGAACAGAAGCAACAGAAGATGGCCCAACAGTCTATGCAAGTGGGTATGGATAATGGTGCTGCTGCGCTGGCTCAAGGTATGGCTGCACAAGCTACAGCTTCACCTGAGGCTATGgctcc                                                                                                                                                                                                                            >  1:198661/1‑299 (MQ=255)`  
                                                                               `cATTAGTACAGGTCTGGAAGCAATTGGTCGAGGACAAGACCTTGATAAGCTGGAGCGGTGTGTCACTGCGTGGGCTGCACTGGCACCTATGCGGGACGACCCTGATATTAACCTTGCGATGATTAAGTTACGTATTGCCAACGCTATCGGTATTGACACTTCTGGTATTCTACTCACCGAAGAACAGAAGCAACAGAAGATGGCCCAACAGTCTATGCAAGTGGGTATGGATAATGGTGCTGCTGCGCTGGCTCAAGGTATGGCTGCACAAGCTACAGCTTCACCTGAGGCTATGgctgct                                                                                                                                                                                                                           >  1:24791/1‑301 (MQ=255)`  
                                                                                  `tAGTACAGGTCTGGAAGCAATTAGTCGAGGACAAGACCTTGATAAGCTGGAGCGGTGTGTCACTGCGTGGGCTGCACAGGCACCTATGCGGGACGACACTGATTTTAACCTTGCGATGATTAAGTTACGTATTGCTAACGCTATCGGTATTGACACTTCTGTTATTCTTCTCACCGAAGAACAGAATCAACAGAAGATGTCCCAACAGTATATGCAATTGGGTATGGATAATGGTGCGTCTGCGCTGGCTTAAGTTATGGCTGCAAAACCTACAGCTTCATCTGAGGCTATGGCtgctgcg                                                                                                                                                                                                                        >  1:347462/1‑300 (MQ=255)`  
                                                                                                        `tgtataagagacagccTGGATAAGCTGGAGCGGTGTGTCACTGCGTGGGCTGCACTGGCACCTATGCGGGACGACCCTGATATTAACCTTGCGATGATTAAGTTACGTATTGCCAACGCTATCGGTATTGACACTTCTGGTATTCTACTCACCGAACAACAGAAGCAACAGAAGATGGCCCAACAGTCTATGCAAGTGGGTATGGATAATGGTGCTGCTGCGCTGGCTCAAGGTATGGCTGCACAAGCTACAGCTTCACCTGAGGCTATGGCTGCTGCCGCTGATTCCGTAGGTTTACAGc                                                                                                                                                                                                  <  1:278983/287‑1 (MQ=255)`  
                                                                                                            `agatgtgtataagagacaggCTGGAGCGGTGTGTCACTGCGTGGGCTGCACTGGCACCTATGCGGGACGACCCTGATATTAACCTTGCGATGATTAAGTTACGTATTGCCAACGCTATCGATATTGACACTTCTGGTATTCTACTCACCGAAGAACAGAAGCAACAGAAGATGGCCCAACAGTCTATGCAAATGGGTATGGATAATGGTGCTGCTGCGCTGGCTCAAGGTATGGCTGCACAAGCTACAGCTTCACCTGAGGCTATGGCTGCTGCCGCTGATTCCGTAGGTTTACAGCCggg                                                                                                                                                                                              <  1:3948/282‑1 (MQ=255)`  
                                                                                                                     `aCCTTGATAAGCTGGAGCGGTGTGTCACTGCGTGGGCTGCACTGGCACCTATGCGGGACGACCCTGATATTAACCTTGCGATGATTAAGTTACGTATTGCCAACGCTATCGGTATTGACACTTCTGGTATTCTACTCACCGAAGAACAGAAGCAACAGAAGATGGCCCAACAGTCTATGCAAGTGGGTATGGATAATGGTGCTGCTGCGCTGGCTCAAGGTATGGCTGCACAAGCTACAGCTTCACCTGAGGCTATGGCTGCTGCCGCTGATTCCGTAGGTTTACAGCCGGGAATTTAATa                                                                                                                                                                                     >  1:47801/1‑301 (MQ=255)`  
                                                                                                                      `ccTTGATAAGCTGGAGCGGTGTGTCACTGCGTGGGCTGCACTGGCACCTATGCGGGACGACCCTGATATTAACCTTGCGATGATTAAGTTACTTATTGCCAACGCTATCGGTATTGACACTTCTGGTATTCTACTCACCGAAGAACAGAAGCAACAGAAGATGGCCCAACAGTCTATGCAAGTGGGTATGGATAATGGTGCTGCTGCGCTGGCTCAAGGTATGGCTGCACAAGCTACAGCTTCACCTGAGGCTATGGCTGCTGCCGCTGATTCCGTAGGTTTACAGCCGGGAATTTAATAc                                                                                                                                                                                    <  1:174128/301‑1 (MQ=255)`  
                                                                                                                         `tGATAAGCTGGAGCGGTGTGTCACTGCGTGGGCTGCACTGGCACCTATGCGGGACGACCCTGATATTAACCTTGCGATGATTAAGTTACGTATTGCCAACGCTATCGGTATTGACACTTCTGGTATTCTACTCACCGAAGAACAGAAGCAACAGAAGATGGCCCAACAGTCTATGCAAGTGGGTATGGATAATGGTGCTGCTGCGCTGGCTCAAGGAATGGTTGCACAAGCTAAAGCTTCACCTGAGGCTATGGCTGCTGCCGCTGATTCAGTAGGTTTACAGCCGGGAATTTAATACGaa                                                                                                                                                                                 >  1:287671/1‑300 (MQ=255)`  
                                                                                                                         `tGATAAGCTGGAGCGGTGTGTCACTGCGTGGGCTGCACTGGCACCTATGCGGGACGACCCTGATATTAACCTTGCGATGAGTAATTTACGTATTGCCAACGCTATCGGTCTTGACACTTCTGGTTTTCTCCTCACCGCAGAACAGCAGCACCAGAAGCTGGCCCAACAGTCTATGCACGTGGGTATGGATAATGGTGCTGCTGCGCTGGCTCAAGGTATGGCTGCACAAGCTACAGCTTCACCGGAGGCTATGGCTGCTGCCGCTGATTCCGTAGGTGTACAGCCGGGAATTTAATACGAc                                                                                                                                                                                 >  1:236924/1‑301 (MQ=255)`  
                                                                                                                         `ggATAAGCTGGATCGGTGTGTCACTGCGTTGGCTTGACTGGCACCTATGCGGGACGACCCTGATATTAACCTTGCGATTATTAAGTTACGTATTGCCAACGCTATCTGTATTGACACTTCTGGTATGCTACTCACCGAAGAACAGAAGCAACAGAAGATGGCCCAACAGTCTATGCAAGTGGGTATGGATAATGGTGCTGCTGCGCTGGCTCAAGGTATGGCTGCACAAGCTACAGCTTCACCTGAGGCTATGGCTGCTGCCGCTGATTCCGTAGGTTTACAGCCGGGAATTTAATACGAc                                                                                                                                                                                 <  1:78687/300‑1 (MQ=255)`  
                                                                                                                          `gATAAGCTGGAGCGGTGTGTCACTGCGTGGGCTGCACTGGCACCTATGCGGGACGACCCTGATATTAACCTTGCGATGATTAAGTTACGTATTGCCAACGCTATCGGTATTGACACTTCTGGTATTCTACTCACCGAAGAACAGAAGCAACAGAAGATGGCCCAACAGTCTATGCAAGTGGGTATGGATAATGGTGCTGCTGCGCTGGCTCAAGGTATGGCTGCACAAGCTACAGCTTCACCTGCGGCTATGGCTGCTGCCGCTGATTCCGTAGGTTTACAGctgtctcttatacacatct                                                                                                                                                                                >  1:167629/1‑283 (MQ=255)`  
                                                                                                                              `aGCTGGAGCGGTGTGTCACTGCGTGGGCTGCACTGGCACCTATGCGGGACGACCCTGATATTAACCTTGCGATGATTAAGTTACGTATTGCCAACGCTATCGGTATTGACACTTCTGGTATTCTACTCACCGAAGAACAGAAGCAACAGAAGATGGCCCAACAGTCTATGCAAGTGGGTATGGATAATGGTGCTGCTGCGCTGGCTCAAGGTATGGCTGCACAAGCTACAGCTTCACCTGAGGCTATGGCTGCTGCCGCTGATTCCGTAGGTTTACAGCCGGGAATTTAATACGACTCACt                                                                                                                                                                            >  1:54018/1‑301 (MQ=255)`  
                                                                                                                                `cTGGAGCGGTGGGTCACTGCGTGGGCTGCACTGGCACCTATGCGGGACGACCCTGATATTAACCTTGCGATGATTAAGTTACGTATTGCCAACGCTATCGGTATTGACACTTCTGGTATTCTACTCACCGAAGAACAGAAGCAACAGAAGATGGCCCAACAGTCTATGCAAGTGGGTATGGATAATGGTGCTGCTGCGCTGGCTCAAGGTATGGCTGCACAAGCTACAGCTTCACCTGAGGCTATGGCTGCTGCCGCTGATTCCGTAGGTTTACAGCCGGGAATTTAATACGACTCACtat                                                                                                                                                                          <  1:276594/301‑1 (MQ=255)`  
                                                                                                                                `aaagagacaggtgtCAATGCGTGGGCTGCACTGGCACCTATGCGGGACGACCCTGATATTAACCTTGCGATGATTAAGTTACGTATTGCCAACGCTATCGGTATTGACACTTCTGGTATTCTACTCACCGAAGAACAGAAGCAACAGAAGATGGCCCAACAGTCTATGCAAGTGGGTATGGATAATGGTGCTGCTGCGCTGGCTCAAGGTATGGCTGCACAAGCTACAGCTTCACCTGAGGCTATGGCTGCTGCCGCTGATTCCGTAGGTTTACAGCCGGGAATTTAATACGACTCACtat                                                                                                                                                                          <  1:171184/291‑1 (MQ=255)`  
                                                                                                                                  `ggAGTGGTGTGTCACTGCGTGGGCTGCACTGGCACCTATGCGGGACGACCCTGATATTAACCTTGCGATGAGTAAGTTACGTATTGCCAACGCTATCGGTATTGGCACTTCTGGTATTCTACTCACCGAAGAACAGAAGCAACAGAAGATGGCCCAACAGTCTATGCAAGTGGGTATGGATAATGGTGCGGCTGCGCTGGCTCAAGGTATGGCTGCACAAGCTAAAGCTTAACCTGAGGCTATGGCTGCTGCCACTGATTCCGTAGGTTACCAGCCGGGAATCTAATAGGACTCACTATAg                                                                                                                                                                        >  1:121296/1‑301 (MQ=255)`  
                                                                                                                                  `ggAGCGGTGTGTCACTGCGTGGGCTGCACTGGCACCTATGCGGGACGACCCTGATATTAACCTTGCGATGATTAAGTTACGTATTGCCAACGCTATCGGTATTGACACTTCTGGTATTCTACTCACCGAAGAACAGAAGCAACAGAAGATGGCCCAACAGTCTATGCAAGTGGGTATGGATAATGGTGCTGCTGCGCTGGCTCAAGGTATGGCTGCACAAGCTACAGCTTCACCTGAGGCTATGGCTGCTGCCGCTGATTCCGTAGGTTTACAGCCGGGCATTTAATACGACTCACTATAg                                                                                                                                                                        >  1:27178/1‑301 (MQ=255)`  
                                                                                                                                   `gAGCGGTGTGTCACTGCGTGGGCTGCACTGGCACCTATGCGGGACGACCCTGATATTAACCTTGCGATGATTAAGTTACGTATTGCCAACGCTATCGGTATTGACACTTCTGGTATTCTACTCACCGAAGAACAGAAGCAACAGAAGATGGCCCAACAGTCTATGCAAGTGGGTATGGATAATGGTGCTGCTGCGCTGGCTCAAGGTATGGCTGCACAAGCTACAGCTTCACCTGAGGCTATGGCTGCTGCCGCTGATTCCGTAGGTTTACAGCCGGGAATTTAATACGACTCACTATAgg                                                                                                                                                                       <  1:24654/301‑1 (MQ=255)`  
                                                                                                                                    `tgtataagagacaggCGTGGGCTGCACTGGCACCTATGCGGGACGACCCTGATATTAACCTTGCGATGATTAAGTTACGTATTGCCAACGCTATCGGTATTGACACTTCTGGTATTCTACTCACCGAAGAACAGAAGCAACAGAAGATGGCCCAACAGTCTATGCAAGTGGGTATGGATAATGGTGCTGCTGCGCTGGCTCAAGGTATGGCTGCACAAGCTACATCTTCACCTGAGGCTATGGCTGCTGCCGCTGATTCCGTAGGTTTACAGCCGGGAATTTAATACGACTCACTATAggg                                                                                                                                                                      <  1:117080/287‑1 (MQ=255)`  
                                                                                                                                    `ggtgtataagagacaggTGGGCTGCACTGGCACCTATGCGGGACGACCCTGATATTAACCTTGCGAGGATGAAGTTACGTATTGCCAACGATATCGGTATTGACACTTCTGGTATTCTACTCACCGAAGAACAGAAGCAACAGAAGATGGCCCAACAGTCTATGCAAGTGGGTATGGATAATGGTGCTGCTGCGCTGGCTCAAGGTATGGCTGCACAAGCTACAGCTTCACCTGAGGCTATGGCTGCTGCCGCTGATTCCGTAGGTTTACAGCCGGGAATTTAATACGACTCACTATAggg                                                                                                                                                                      <  1:316594/285‑1 (MQ=255)`  
                                                                                                                                    `ggCGGTGGGTCACTGCGTGGGCTGCACTGGCACCTATGCGGGACGACCCTGATATTAAACTTGCGATGATTAAGTTACGTATTGCCAACGCTATCGGTATTGACACTTCTGGTATTCTACTCACCGAAGAACAGAAGCAACAGAAGATGGCCCAACAGTCTATGCAAGTGGGTATGGATAATGGTGCTGCTGCGCTGGCTCAAGGTATGGCTGCACAAGCTACAGCTTCACCTGAGGCTATGGCTGCTGCCGCTGATTCCGTAGGTTTACAGCCGGGAATTTAATACGACTCACTATAggg                                                                                                                                                                      <  1:219619/300‑1 (MQ=255)`  
                                                                                                                                    `agacaggtgtCACTGCGTGGGCTGCACTGGCACCTATGCGGGACGACCCTGATATTAACCTTGCGATGATTAAGTTACGTATTGCCAACGCTATCGGTATTGACACTTCTGGTATTCTACTCACCGAAGAACAGAAGCAACAGAAGATGGCCCAACAGTCTATGCAAGTGGGTATGGATAATGGTGCTGCTGCGCTGGCTCAAGGTATGGCTGCACAAGCTACAGCTTCACCTGAGGCTATGGCTGCTGCCGCTGATTCCGTAGGTTTACAGCCGGGAATTTAATACGACTCACTATAggg                                                                                                                                                                      <  1:293682/295‑1 (MQ=255)`  
                                                                                                                                    `acaggtggttCACTGCGTGGGCTGCACTGGCACCTATGCGGGACGACCCTGATATTAACCTTGCGATGATTAAGTTACGTATTGCCAACGCTATCGGTATTGACACTTCTGGTATTCTACTCACCGAAGAACAGAAGCAACAGAAGATGGCCCAACAGTCTATGCAAGTGGGTATGGATAATGGTGCTGCTGCGCTGGCTCAAGGTATGGCTGCACAAGCTACAGCTTCACCTGAGGCTATGGCTGCTGCCGCTGATTCCGTAGGTTTACAGCCGGGAATTTAATACGACTCACTATAggg                                                                                                                                                                      <  1:282050/292‑1 (MQ=255)`  
                                                                                                                                     `gCGGTGTGTCACTGCGTGGGCTGCACTGGCACCTATGCGGGACGACCCTGATATTAACCTTGCGATGATTAAGTTACGTATTGCCAACGCTATCGGTATTGACACTTCTGGTATTCTACTAACCGAAGAACAGAAGAAACAGAAGATGGCCCAACAGTCTATGCAAGTGGGGATGGATAATGGTGCTGCTGCGCTGGCTCAAGGTATGGCTGCACAAGCTACAGCTTCACCTGAGGCGATGGCTGCTGCCGCTGATTCCGTAGGTTTACAGCCGGGAATTTAATACGACTCACTATAGGga                                                                                                                                                                     <  1:333327/301‑1 (MQ=255)`  
                                                                                                                                     `gCGGTGTGTCACTGCGTGGGCTGCACTGGCACCTATGCGGGACGACCATGATATTAACCTTGCGATGATTAAGTTACGTATTGCCAACGCTATCGGTATTGACACTTCTGGTATTCTATTCACCGAAGAACAGAAGCAACAGAAGATGGCCCAACAGTCTATGCAAGTGGGTATGGATAATGGTGCTGCTGCGCTGGCTCAAGGTATGGCTGCACAAGCTACAGCTTCACCTGAGGCTATGGCTGCTGCCGCTGATTCCGTAGGTTTACAGCCGGGAATTTAATACGACTCACTATAGGga                                                                                                                                                                     <  1:325986/301‑1 (MQ=255)`  
                                                                                                                                      `agacaggtCACTGCGTGGGCTGCACTGGCACCTATGCGGGACGACCCTGATATTAACCTGGCGATGATTAAGTTACGTATTGCCAACGCTATCGGTATTGACACTTCTGGTATTCTACTCACCGAAGAACAGAAGCAACAGAAGATGGCCCAACAGTCTATGCAAGTGGGTATGGATAATGGTGCTGCTGCGCTGGCTCAAGGTATGGCTGCACAAGCTACAGCTTCACCTGAGGCTATGGCTGCTGCCGCTGATTCCGTAGGTTTACAGCCGGGAATTTAATACGACTCACTATAGGgag                                                                                                                                                                    <  1:250433/295‑1 (MQ=255)`  
                                                                                                                                      `acaggtgtCACTGCGTGGGCGTCACTGGCACCTATGCGGGACGACCCTGATATTAACCTTGCGATGATTAAGTTACGTATTGCCAACGCTATCGGTATTGACACTTCTGGTATTCTACTCACCGAAGAACAGAAGCAACAGAAGATGGCCCAACAGTCTATGCAAGTGGGTATGGATAATGGTGCTGCTGCGCTGGCTCAAGGTATGGCTGCACAAGCTACAGCTTCACCTGAGGCTATGGCTGCTGCCGCTGATTCCGTAGGTTTACAGCCGGGAATTTAATACGACTCACTATAGGgag                                                                                                                                                                    <  1:329531/297‑1 (MQ=255)`  
                                                                                                                                        `gtgtgtCACTGCGTGGGCTGCACTGGCACCTATGCGGGACGACCCTGATATTAACCTTGCGATGATTAAGTTACGTATTGCCAACGCTATCGGTATTGACACTTCTGGTATTCTACTCACCGAAGAACAGAAGCAACAGAAGATGGCCCAACAGTCTATGCAAGTGGGTATGGATAATGGTGCTGCTGCGCTGGCTCAAGGTATGGCTGCACAAGCTACAGCTTCACCTGAGGCTATGGCTGCTGCCGCTGATTCCGTAGGTTTACAGCCGGGAATTTAATACGACTCACTATAgggctgg                                                                                                                                                                  >  1:239890/1‑297 (MQ=255)`  
                                                                                                                                          `gtgtCACTGCGTGGGCTGCACTGGCACCTATGCGGGACGACCCTGATATTAACCTTGCGATGATTAAGTTACGTATTGCCAACGCTATCGGTATTGACACTTCTGGTATTCTACTCACCGAAGAACAGAAGCAACAGCAGATGGCCCAACAGTCTATGCAAGTGGGTATGGATAATGGTGCTGCTGCGCTGGCTCAAGGTATGGCTGCACAAGCTACAGCTTCACCTGAGGCTATGGCTGCTGCCGCTGATTCCGTAGGTTTACAGCCGGGAATTTAATACGACTCACTATAGGGAGACCt                                                                                                                                                                >  1:144742/1‑301 (MQ=255)`  
                                                                                                                                          `gtgtCACTGCGTGGGCTGCACTGGCACCTATGCGGGACGACCCTGATATTAACCTTGCGATGATTAAGTTACGTATTGCCAACGCTATCGGTATTGACACTTCTGGTATTCTACTCACCGAAGAACAGAAGCAACAGAAGATGGCCCAACAGTCTATGCAAAGGGGTATGGATAATGGTGCTGCTGCGCTGGCTCAAGGTATGGCTGCACAAGCTACAGATTCACCTGAGGCTATGGCTGCTGCCGCTGATTCCGTAGGTTTACAGCCGGGAATTTAATACGACTCACTATAGGGAGACCt                                                                                                                                                                >  1:50901/1‑301 (MQ=255)`  
                                                                                                                                          `gtgtCACTGCGTGGGCTGCACTGGCACCTATGCGGGACGACCCTGATATTAACCTTGCGATGATTAAGTTACGTATTACCAACGCTATCGGTATTGACACTTCTGGTATTCTACTCACCGAAGAACAGAAGCAACAGAAGATGTCCCAACAGTCTATGCAAGTGGGTATGGATAATGGTGCTGCTGCGCTGGCTCAAGGTATGGCTGCACAAGCTACAGCTTCACCTGAGGCTATGGCTGCTGCCGCTGATTCCGTAGGTTTACAGCCGGGAATTTAATACGACTCACTATA‑GGAGACCTc                                                                                                                                                               >  1:119223/1‑301 (MQ=255)`  
                                                                                                                                                    `gTGGGCTGCACTGGCACCTATGCGGGACGACCCTGATATTAACCTTTCGATGATTAAGTTACGTATTGCCAACGCTATCGGTATTGACACTTCTGGTATTCTACTCACCGAAGAACAGAAGCAACAGAAGATGGCCCAACAGTCTATGCAAGTGGGTATGGATAATGGTGCTGCTGCGCTGGCTCAAGGTATGGCTGCACAAGCTCCAGCTTCACCTGAGGCTATGGCTGCTGCCGCTGATTCCGTAGGTTTACAGCCGGGAATTTACTACGACTCACTATAgggctgtctcttatacaca                                                                                                                                                      >  1:84384/1‑285 (MQ=255)`  
                                                                                                                                                           `gCACTGGCACCTATGCGGGACGACCCTGATATTAACCTTGCGATGATTAAGTTACGTATTGCCAACGCTATCGGTGTTGACACTTCTGGTATTCTACTCACCGAAGAACAGAAGCAACAGAAGATGGCCCAACAGTCTATGCAAGTGGATATGGATAATGGTGCTGCTGCGCTGGCTCAAGGTATGGCTGCACAAGCTACAGCTTCACCTGAGGCTATGGCTGCTGCCGCTGATTCCGTAGGTTTACAGCCGGGAATTTAATACGACTCACTATAGGGATACCTCATCTTTGAAATGAGCg                                                                                                                                               >  1:286050/1‑301 (MQ=255)`  
                                                                                                                                                           `gCACTGGCACCTATGCGGGACGACCCTGATATTAACCTTGCGATGATTAAGTTACGTATTGCCAACGCTATCGGTATTGACACTTCTGGTATTCTACTCACCGAAGAACAGAAGCAACAGAAGATGGCCCAACAGTCTATGCAAGTGGGTATGGATAATGGTGCTGCTGCGCTGGCTCAAGGTATGGCTGCACAAGCTACAGCTTCACCTGAGGCTATGGCTGCTGCCGCTGATTCCGTAGGTTTACAGCCGGGAATTTAATACGACTCACTATAGGGAGACCTCATCTTTGAAATGAGCg                                                                                                                                               >  1:253675/1‑301 (MQ=255)`  
                                                                                                                                                                     `gagacagggACGACCCTGATATTAACCTTGCGATGATTAAGTTACGTATTGCCAACGCTATCGGTATTGACACTTCTGGTATTCTACTCACCGAAGAACAGAAGCAACAGAAGATGGCCCAACAGTCTATGCAAGTGGGTATGGATAATGGTGCTGCTGCGCTGGCTCAAGGTATGGCTGCACAAGCTACAGCTTCACCTGAGGCTATGGCTGCTGCCGCTGATTCCGTAGGTTTACAGCCGGGAATTTAATACGACTCACTATAGGGAGACCTCATCTTTGAAATGAGCGATGACAagag                                                                                                                                     <  1:42652/295‑1 (MQ=255)`  
                                                                                                                                                                      `gggctcggagatgtgtataagagacagtGCGATGATTAAGTTACGTATTGCCAACGCTATCGGTATTGACACTTCTGGTATTCTACTCACCGAAGAACAGAAGCAACAGAAGATGGCCCAACAGTCTATGCAAGTGGGTATGGATAATGGTGCTGCTGCGCGGGCTCAAGGTATGGCTGCACAAGCTACAGCTTCACCTGAGGCTATGGCTGCTGCCGCTGATTCCGTAGGTTTACAGCCGGGAATTTAATACGACTCACTATAGGGAGACCTCATCTTTGAAATGAGCGATGACAAGAgg                                                                                                                                    <  1:337472/274‑1 (MQ=255)`  
                                                                                                                                                                       `aTGCGGGACGACCCTGATATTAACCTTGCGATGATTAAGTTACGTATTGCCAACGCTATCGGTATTGACACTTCTGGTATTCTACTCACCGAAGAACAGAAGCAACAGAAGATGGCCCAACAGTCTATGCAAGTGGGTATGGATAATGGTGCTGCTGCGCTGGCTCAAGGTATGGCTGCACAAGCTACAGCTTCACCTGAGGCTATGGCTGCTGCCGCTGATTCCGTAGGTTTACAGCCGGGAATTTAATACGACTCACTATAGGGAGACCTCATCTTTGAAATGAGCGATGACAAGAGGt                                                                                                                                   >  1:59277/1‑301 (MQ=255)`  
                                                                                                                                                                       `aTGCGGGACGACCCTGATATTAACCTTGCGATGATTAAGTTACGTATTGCCAACGCTATCGGTATTGACACTTCTGGTATTCTACTCACCGAAGAACAGAAGCAACAGAAGATGGCCCAACAGTCTATGCAAGTGGGTATGGATAATGGTGCTGCTGCGCTGGCTCAAGGTATGGCTGCACAAGCTACAGCTTCACCTGAGGCTATGGCTGCTGCCGCTGATTCCGTAGGTTTACAGCCGGGAATTTAATACGACTCACTATAGGGAGACCTCATCTTTGAAATGAGCGATGACAAGAGGt                                                                                                                                   >  1:18509/1‑301 (MQ=255)`  
                                                                                                                                                                            `taagagacaggattgtAACCTTGCGATGATTAAGTTACGTATTGCCAACGCCATCGGTATTGACACTTCTGGTATTCTACTCACCGAAGAACAGAAGCAACAGAAGATGGCCCAACAGTCTATGCAAGTGGGTATGGATAATGGTGCTGCTGCGCTGGCTCAAGGTATGGCTGCACAAGCTACAGCTTCACCTGAGGCTATGGCTGCTGCCGCTGATTCCGTAGGGTTACAGCCGGGAATTTAATACGACTCACTATAGGGAGACCTCATCTTTGAAATGAGCGATGACAAGAGGTTGGAg                                                                                                                              <  1:153809/286‑1 (MQ=255)`  
                                                                                                                                                                            `gacagacCCTGATATTAACCTTGCGATGATGAAGTGACGTAGTGCCAACGCTAGCGGTATTGACACTTCTGGTATTCTACTCACCGAAGAACAGAAGCAACAGAAGATGGCCCAACAGTCTATGCAAGTGGGTATGGATAATGGAGCTGCTGCGCTGGCTCAAGGTATGGCTGCACAAGCTACAGCTTCACCTGAGGCTATGGCTGCTGCCGCTGATTCCGTAGGTTTACAGCCGGTAATTTAATACGACTCACTATAGGGAGACCTCATCTTTGAAATGAGCGATGACAAGAGGTTGGAg                                                                                                                              <  1:195049/297‑1 (MQ=255)`  
                                                                                                                                                                            `aaagagataggaaatTAACCAGTCGATGATTAAGTTACAAGTTGCCAACGCTAGCTGTATTGACAGTTCTGGTATTCTACTCACCGAAGAACAAAAGCAACAGAAGATGGCCAAACATTCTATGCAAGTGGGTATGGATAATGGTGCTGCGGCGCTGGCTCAAGGTAAGGCGGCACAAGCTGCAGCTTCACCTGAGACTATAGCTGCTGCCGATGATTCCGTAGGTGTACAGCCGGGAGGTAAATACGCGTCACTATAGGGAGAACTCATCTTTGAAATGAGCGATGACAAGAGGTTGGAg                                                                                                                              <  1:151263/288‑1 (MQ=255)`  
                                                                                                                                                                              `acgacCCTGATATTAACCTTGCGATGATTAAGTTACGTATTGCCAACGCTATCGGTATTGACACTTCTGGTATTCTACTCACCGAAGAACAGAAGCAACAGAAGATGGCCCAACAGTCTATGCAATTGGGTATGGATAATGGTGCTGCTGCGCTGGCTCAAGGTATGGCTGCACAAGCTACAGCTTCACCTGAGGCTATGGCTGCTGCCGCTGATTCCGTAGGTTTACAGCCGGGAATTTAATACGACTCACTATAGGGAGACCTCATCTTTGAAATGAGCGATGACAAGAGGTTGGAGTc                                                                                                                            <  1:265975/301‑1 (MQ=255)`  
                                                                                                                                                                                 `acCCTGATCTTAACCTTGCGATGATTAAGTTACGTATTGCCAACGCTATCGGTATTGACACTTCTGGTATTCTACTCACCGAAGAACAGAAGCAACAGAAGATGGCCCAACAGTCTATGCAAGTGGGTATGGATAATGGTGCTGCTGCGCTGGCTCAAGGTATGGCTGCACAAGCTACAGCTTCACCTGAGGCTATGGCTGCTGCCGCTGATTCCGTAGGTTTACAGCCGGGAATTTAATACGACTCATTATAGGGAGACCTCATCTTTGAAATGAGCGATGACAAGAGGTTGGAGTCCTc                                                                                                                         >  1:233868/1‑301 (MQ=255)`  
                                                                                                                                                                                 `acCCTGATATTAACCTTGCGATGATTAAGTTACGTATTGCCAACGCTATCGGTATTGACACTTCTGGTATTCTACTCACCGAAGAACAGAAGCAACAGAAGATGGCCCAACAGTCTATGCAAGTGGGTATGGATAATGGTGCTGCTGCGCTGGCTCAAGGTATGGCTGCACAAGCTACAGCTTCACCTGAGGCTATGGCTGCTGCCGCTGATTCCGTAGGTTTACAGCCGGGAATTTAATACGACTCACTATAGGGAGACCTCATCTTTGAAATGAGCGATGACAAGAGGTTGGAGTCCTc                                                                                                                         >  1:187401/1‑301 (MQ=255)`  
                                                                                                                                                                                      `gATATTAACCTTGCGATGATTAAGTTACGTATTGCCAACGCTATCGGTATTGACACTTCTGGTATTCTACTCCCCGAAGAACAGAAGCAACAGAAGATGGCCCAACAGTCTATGCAAGTGGGTATGGATAATGGTGCTGCTGCGCTGGCTCAAGGTATGGCTGCACAAGCTACAGCTTCACCTGAGGCTATGGCTGCTGCCGCTGATTCCGTAGGTTTACAGCCGGGAATTTAATACGACTCACTATAGGGCGACCTCATCTTTGAAATGAGCGATGACAAGAGGCTgtctcttatacaca                                                                                                                    >  1:212145/1‑288 (MQ=255)`  
                                                                                                                                                                                      `gATATTAACCTTGCGATGATTAAGTTACGTATTGCCAACGCTATCGGTATTGACACTTCTGGTATTCTACTCACCGAAGAACAGAAGCAACAGAAGATGGCCCAACAGTCTATGCAAGTGGGTATGGATAATGGTGCTGCTGCGCTGGCTCAAGGTATGGCTGCACAAGCTACAGCTTCACCTGAGGCTATGGCTGCTGCCGCTGATTCCGTAGGTTTACAGCTGGGAATTGAATACGACTCACTATAGGGAGACCTCATCTTTGAAATGAGCGATGACAAGAGGTTGGAGTCCTCGGTCt                                                                                                                    >  1:300804/1‑301 (MQ=255)`  
                                                                                                                                                                                      `gATATTAACCTTGCGATGATTAAGTTACGTATTGCCAACGCTATCGGTATTGACACTTCTGGTATTCTACTCACCGAAGAACAGAAGCAACAGAAGATGGCCCAACAGTCTATGCAAGTGGGTATGGATAATGGTGCTGCTGCGCTGGCTCAAGGTATGGCTGCACAAGCTACAGCTTCACCTGAGGCTATGGCTGCTGCCGCTGATTCCGTAGGTTTACAGCCGGGAATTTAATACGACTCACTATAGGGAGACCTCATCTTTGAAATGAGCGATGACAAGAGGTTGGAGTCCTCGGTcc                                                                                                                    >  1:114096/1‑300 (MQ=255)`  
                                                                                                                                                                                      `gATATTAACCTTGCGATGATTAAGTTACGTATTGCCAACGCTATCGGTATTGACACTTCTGGTATTCTACTCACCGAAGAACAGAAGCAACAGAAGATGGCCCAACAGTCTATGCAAGTGGGTATGGATAATGGTGCTGCTGCGCTGGCTCAAGGTATGGCTGCACAAGCTACAGCTTCACCTGAGGCTATGGCTGCTGCCGCTGATTCCGTAGGTTTACAGCCGGGAATTTAATACGACTCACAATAGGGAGACCTCATCTTTGAAATGAGCGATGACAAGAGGTTGGAGTCCTCGGTCt                                                                                                                    <  1:92203/301‑1 (MQ=255)`  
                                                                                                                                                                                       `atatTAACCTTGCGATGATTAAGTTACGTATTGCCAACGCTATCGGTATTGACACTTCTGGTATTCTACTCACCGAAGAACAGAAGCAACAGAAGATGGCCCAACAGTCTATGCAAGTTGTTGTGGATAATGGTGCTGCTGCGCTGGCTCAAGGTATGGCAGCACAAGCTACATCTTCACCTGAGGCTATGGCTGCTGCCGCTGAGTCCGTAGTTTTACAGCCGGGAATTCATAAAGACTCACTATATGGATACCTCATTTTTGAAATAAGCGATGACAATCGGTTTGAGTCCTACGTCtt                                                                                                                   >  1:69507/1‑301 (MQ=255)`  
                                                                                                                                                                                            `aaCCTTGCGATGATTAAGTTACGTATTGCCAACGCTATCGGTATTGACACTTCTGGTATTCTACTCACCGAAGAACAGAAGCAACAGAAGATGGCCCAACAGTCTATGCAAGTGGGTATGGATAATGGTGATGCTGCGCTGGCTCAAGGTATGGCTGCACAAGCTACAGCTTCACCTGAGGCTATGGATGCTGCCGCTGATTCCGTAGGTTTACAGCCGGGAATTTAATACGACTCACTATAGGGAGACCTCATCTTTGAAATGAGCGATGACAAGAGGTTGGAGTCCTCGGTCTTCCTGt                                                                                                              <  1:209220/301‑1 (MQ=255)`  
                                                                                                                                                                                              `cgtgggctcggagatgtgtataagagacagcGCTATCGGTATTGACACTTCTGGTATTCTACTCACCGAAGAACAGAAGCAACAGAAGATGGCCCAACAGTCTATGCAAGTGGGTATGGATAATGGTGCTGCTGCGCTGGCTCAAGGTATGGCTGCACAAGCTACAGCTTCACCTGAGGCTATGGCTGCTGCCGCTGATTCCGTAGGTTTACAGCCGGGAATTTAATACGACTCACTATAGGGAGACCTCATCTTTGAAATGAGCGATGACAAGAGGTTGGAGTCCTCGGTCTTCCTGTAg                                                                                                            <  1:319759/271‑1 (MQ=255)`  
                                                                                                                                                                                                                     `ttGCCAACGCTCTCGGTATTGACACTTCTGTGATTCTCCTCACCGAAGAACAGAAGCAACAGAAGAGGGCCCAACAGTCTATGCAAGTGGGGATGGATAATGGTGCTGCTGCGCTGGCTCAAGGTATGGCTGCACAAGCTACAGCTTCACCTGAGGCTATGGCTGCTGCCGCTGATTCCGTAGGTTTACAGCCGGGAATTTAATACGACTCACTATAGGGAGACCTCATCTTTGAAATGAGCGATGACAAGAGGTTGGAGTCCTCGGTCTTCCTGTAGTTCAACTTTAAGGAGACaataat                                                                                     <  1:103272/301‑1 (MQ=255)`  
                                                                                                                                                                                                                     `ttGCCAACGCTATCGGTATTGACACTGCTGGTATTCTACTCACCGAAGAACAGAAGCAACAGAAGATGGCCCAACAGTCTATGCAAGTGGGTATGGATAATGGTGCTGCTGCGCTGGCTCAAGGTATGGCTGCACAAGCTACAGCTTCACCTGAGGCTATGGCTGCTGCCGCTGATTCCGTAGGTTTACAGCCGGGAATTTAATACGACTCACTATAGGGAGACCTCATCTTTGAAATGAGCGATGACAAGAGGTTGGAGTCCTCGGTCTTCCTGTAGTTCAACTTTAAGGAGACaataat                                                                                     <  1:268405/301‑1 (MQ=255)`  
                                                                                                                                                                                                                     `ctcggagatgtgtataagagacagcTTCTGGTATTCTACTCACCGAAGAACAGAAGCAACAGAAGATGGCCCAACAGTCTATGCAAGTGGGTATGGATAATGGTGCTGCTGCGCTGGCTCAAGGTATGGCTGCACAAGCTACAGCTTCACCTGAGGCTATGGCTGCTGCCGCTGATTCCGTAGGTTTACAGCCGGGAATTTAATACGACTCACTATAGGGAGATCTCATCTTTGAAATGAGCGATGACAAGAGGTTGGAGTCCTCGGTCTTCCTGTAGTTCAACTTTAAGGAGACaataat                                                                                     <  1:87916/277‑1 (MQ=255)`  
                                                                                                                                                                                                                       `gCCAACTCTATCGGTATTGACACTTCTGGTACTATACTCACCGAAGAACAGAAGCAACAGAAGATGGCCCAACAGGCTATGCAAGTGGGTATGGATAATGGTGCTGCTGCGCTGGCTCAAGGTATGGCTGCACAAGCTACAGCTTCACCTGAGGCTATGGCTGCTGCCGCTGATTCCGTAGGTTTACAGCCGGGAATTTAATACGACTCACTATAGGGAGACCTCATCTTTGAAATGAGCGATGACAAGAGGTTGGAGTCCTCGGTCTTCCTGTAGTTCAACTTTAAGGAGACaataataa                                                                                   <  1:221815/301‑1 (MQ=255)`  
                                                                                                                                                                                                                       `gCCAACGCTATCGGTATTGACACTTCTGGTATTCTACTCACCGAAGAACAGAAGCAACAGAAGATGGCCCAACAGTCTATGCAAGTGGGTATGGATAATGGTGCTGCTGCGCTGGCTCAAGGTATGGCTGCACAAGCTACAGCTTCACCTGAGGCTATGGCTGCTGCCGCTGATTCCGTAGGTTTACAGCCGGGAATTTAATACGACTCACTATAGGGAGACCTCATCTTTGAAATGAGCGATGACAAGAGGTTGGAGTCCTCGGTCTTCCTGTAGTTCAACTTTAAGGAGACaataataa                                                                                   <  1:12477/301‑1 (MQ=255)`  
                                                                                                                                                                                                                       `cgtatatgtgtataatagacagcTTCTGGTATTCGACTCACCGAAGAACAGAAGCAACTGAAGATGGCCCAACAGTCTATGCAAGTGGGTATGGATAATGGTGCTGCTGCGCTGGCTCAAGGTATGGCTGCACAAGCTACAGCTTCACCTGAGGCTATGGCTGCTGCCGCTGATTCCGTAGGTTTACAGCCGGGGATTTAATACGACTCACTATAGGGAGACCTCATCTTTGAAATGAGCGATGACAAGAGTTTGGAGTCCTCGGTCTTCCTGTAGTTCAACTTTAAGGAGACaataataa                                                                                   <  1:202049/279‑1 (MQ=255)`  
                                                                                                                                                                                                                         `cAACGCTATCGGTATTGACACTTCTGGTATTCTACTCACCGAAGAACAGAAGCAACAGAAGATGGCCCAACAGTCTATGCAAGTGGGTATGGATAATGGTGCTGCTGCGCTGGCTCAAGGTATGGCTGCACAAGCTACAGCTTCACCTGAGGCTATGGCTGCTGCCGCTGATTCCGTAGGTTTACAGCCGGGAATTTAATACGACTCACTATAGGGAGACCTCATCTTTGAAATGAGCGATGACAAGAGGTTGGAGTCCTCGGTCTTCCTGTAGTTCAACTTTAAGGAGACAATAATAATg                                                                                 <  1:256732/301‑1 (MQ=255)`  
                                                                                                                                                                                                                            `cGCTATTGGTATTGACACTTCTGGTATTCTACTCACCGAAGAACAGAAGCAACAGAAGATGGCCCAACAGTCTATGCAAGTGGGTATGGATAATGGTGCTGCTGCGCTGGCTCAAGGTATGGCTGCACAAGCTACAGCTTCACCTGAGGCTATGGCTGCTGCCGCTGATTCCGTAGGTTTACAGCCGGGAATTTAATACGACTCACTATAGGGAGACCTCATCTTTGAAATGAGCGATGACAAGAGGTTGGAGTCCTCGGTCTTCCTGTAGTTCAACTTTAAGGAGACaataataactgtc                                                                              >  1:312403/1‑296 (MQ=255)`  
                                                                                                                                                                                                                              `cTATCGGTATTGACACTTCTGGTATTCTACTCACCGAAGAACAGAAGCAACAGAAGATGGCCCAACAGTCTATGCAAGTGGGTATGGATAATGGTGCTGCTGCGATGGCGCAAGGTATGGCTGCACAAGCTACAGCTTCACCTGAGGATATGGCTGCTGCCGCTGATTCCGTTGGTTTACAGCCGGGAATTTAATCCGACTCCCTATTGGGCGACCTCTACTTTGACATGAGCGATGACAAGAGGTTTGAGTCCTCGGTCTTCCTGTAGTTCAACTTTACGGTCAAaataataaatgtctt                                                                            >  1:36655/1‑294 (MQ=255)`  
                                                                                                                                                                                                                                       `ttGACACTTCTGGTATTCTACTCACCGAAGAACAGAAGCAACAGAAGATGGCCCAACAGTCTATGCAAGTGGGTATGGATAATGGTGCTGCTGCGCTGGCTCAAGGTATGGCTGCACAAGCTACAGCTTCACCTGAGGCTATGGCTGCTGCCGCTGATTCCGTAGGTTTACAGCCGGGAATTTAATACGACTCACTATAGGGAGACCTCATCTTTGAAATGAGCGATGACAAGAGGTTGGAGTCCTCGGTCTTCCTGTAGTTCAACTTTAAGGAGACAATAATAATGGCTGAATCTAATGc                                                                   <  1:307276/301‑1 (MQ=255)`  
                                                                                                                                                                                                                                       `tataagagacaggTATTCTACTCACCGAAGAACAGAAGCAACAGAAGATGGCCCAACAGTCTATGCAAGTGGGTATGGATAATGGTGCTGCTGCGCTGGCTCAAGGTATGGCTGCACAAGCTACAGCTTCACCTGAGGCTATGGCTGCTGCCGCTGATTCCGTAGGTTTACAGCCGGGAATTTAATACGACTCACTATAGGGAGACCTCATCTTTGAAATGAGCGATGACAAGAGGTTGGAGTCCTCGGTCTTCCTGTAGTTCAACTTTAAGGAGACAATAATAATGGCTGAATCTAATGc                                                                   <  1:72333/290‑1 (MQ=255)`  
                                                                                                                                                                                                                                       `ggtataagagacaaaTTCTACTCACCGAAGAACAGAAGCAACAGAAGATGGCCCAACAGTCTATGCAAGTGGGTATGGATAATGGTGCTGCTGCGCTGGCTCAAGGTATGGCTGCACAAGCTACAGCTTCACCTGAGGCTATGGCTGCTGCCGCTGATTCCGTAGGTTTACAGCCGGGAATTTAATACGACTCACTATAGGGAGACCTCATCTTTGAAATGAGCGATGACAAGAGGTTGGAGTCCTCGGTCTTCCTGTAGTTCAACTTTAAGGAGACAATAATAATGGCTGAATCTAATGc                                                                   <  1:297137/287‑1 (MQ=255)`  
                                                                                                                                                                                                                                            `ccTTCTGGTATTCTACTCACCGAAGAACAGAAGCAACAGAAGATGGCCGAACAGTCTATGCAAGTGGGTATGGATAATGGTGCTGCTGCGCTGGCTCAAGGTATGGCTGCACAAGCTACATCTTCACCTGAGGCTATGGCTGCTGCCGCTGATTCCGTAGGTTTACAGCCGGGAATTTAATACGACTCACTATAGGGAGACCTCATCTTTGAAATGAGCGATGACAAGAGGTTGGAGTCCTCGGTCTTCCTGTAGTTCAACTTTAAGGAGACAATAATAATGGCTGAATCTAATGCAGACg                                                              <  1:217569/300‑1 (MQ=255)`  
                                                                                                                                                                                                                                            `acTTCTGGTATTCTACTCGCCGAAGAACAGAAGCAACAGAAGATGGCCCAACAGTCTATGCAAGTGGGTATGGATAATGGTGCTGCTGCGCTGGCTCAAGGTATGGCTGCACAAGCTACAGCTTCACCTGAGGCTATGGCTGCTGCCGCTGATTCCGTAGGTTTACAGCCGGGAATTTAATACGACTCACTATAGGGAGACCTCATCTTTGAAATGAGCGATGACAAGATGTTGGAGTCCTCGGTCTTCCTGTAGTTCAACTTTAAGGAGACAATAATAATGGCTGAATCTAATGCAGACg                                                              <  1:62113/301‑1 (MQ=255)`  
                                                                                                                                                                                                                                                   `gTATTCTACTCACCGAAGAACAGAAGCAACAGAAGATGGCCCAACAGTCTATGCAAGTGGGTATGGATAATGGTGCTGCTGCGCTGGCTCAAGGTATGGCTGCACAAGCTACAGCTTCACCTGAGGCTATGGCTGCTGCCGCTGTTTCCGTAGGTTTACAGCCGGGAATTTAATACGACTCACTATAGGGAGACCTCATCTTTGAAATGAGCGATGACAAGAGGTTGGAGTCCTCGGTCTTCCTGTAGTTCTACTTTAAGGAGACAATAATAATGGCTGAATCTAATGCAGACGTATATGc                                                       >  1:263788/1‑301 (MQ=255)`  
                                                                                                                                                                                                                                                   `gTATTCTACTCACCGAAGAACAGAAGCAACAGAAGATGGCCCAACAGTCTATGCAAGTGGGTATGGATAATGGTGCTGCTGCGCTGGCTCAAGGTATGGCTGCACAAGCTACAGCTTCACCTGAGGCTATGGCTGCTGCCGCTGATTCCGTAGGTTTACAGCCGGGAATTTAATACGACTCACTATAGGGAGACCTCATCTTTGAAATGAGCGATGACGAGAGGTTGGAGTCCTCGGTCTTCCTGTAGTTCAACTTTAAGGAGACAATAATAATGGCTGAATCTAATGCAGACGTATATGc                                                       <  1:29071/301‑1 (MQ=255)`  
                                                                                                                                                                                                                                                     `aTTCTACTCACCGAAGAACAGAAGCAACAGAAGATGGCCCAACAGTCTATGCAAGTGGGTATGGATAATGGTGCTGCTGCGCTGGCTCAAGGTATGGCTGCACAAGCTACAGCTTCACCTGAGGCTATGGCTGCTGCCGCTGATTCCGTAGGTTTACAGCCGGGAATTTAATACGACTCACTATAGGGAGACCTCATCTTTGAAATGAGCGATGACAAGAGGTTGGAGTCCTCGGTCTTCCTGTAGTTCAACTTTAAGGAGACAATAATAATGGCTGAATCTAATGCAGACGTATATGcct                                                     >  1:260990/1‑299 (MQ=255)`  
                                                                                                                                                                                                                                                                             `aaaCAGAAGATGGCCCAACAGTCTGTGCAAGTGGGTATGGATAATGGTGCTGCTGCGCTGGCTCAAGGTATGGCTGCACAAGCTACAGCTACACCTGAGGCTATGGCTGCTGCCGCTGATTCCGTAGGTTTACAGCCGGGAATTTAATACGACACACTATAGGGAAACCTCATCTTTGAAATGAGCGATGACAAGAGGTTGTAGTCCTCGGTCTTCCTGTAGTTCAACTTTAAGGAGACAATAATAATGGCTGAATCTAATGCAGACGTATATGCATCTTTTGGCGTGAACTCCGCTGTGa                             <  1:158051/300‑1 (MQ=255)`  
                                                                                                                                                                                                                                                                               `aCAGAAGATGGCCCAACAGTCTATGCAAGTGGGTATGGATAATGGTGCTGCTGCGCTGGCTCAAGGTATGGCTGCACAAGCTACAGCTTCACCTGAGGCTATGGCTGCTGCCGCTGATTCCGTAGGTTTACAGCCGGGAATTTAATACGACTCACTATAGGGAGACCTCATCTTTGAAATGAGCGATGACAAGAGGTTGGAGTCCTCGGTCTTCCTGTAGTTCAACTTTAAGGAGACAATAATAATGGCTGAATCTAATGCATACGTATATGCATCTTTTGGCGTGAACTCCGCTGTGA‑Tg                          >  1:134093/1‑301 (MQ=255)`  
                                                                                                                                                                                                                                                                               `aCAGAAGATGGCCCAACAGTCTATGCAAGTGGGTATGGATAATGGTGCTGCTGCGCTGGCTCAAGGTATGGCTGCACAAGCTACAGCTTCACCTGAGGCTATGGCTGCTGCCGCTGATTCCGTAGGTTTACAGCCGGGAATTTAATACGACTCACTATAGGGAGACCTCATCTTTGAAATGAGCGATGACAAGAGGTTGGAGTCCTCGGTCTTCCTGTAGTTCAACTTTAAGGAGACAATAATAATGGCTGAATCTAATGCAGACGTATATGCATCTTTTGGCGTGAACTCCGCTGTGA‑Tg                          >  1:64171/1‑301 (MQ=255)`  
                                                                                                                                                                                                                                                                                      `ataagagacaggTCTATGCAAGTGGGTATGGATAATGGTGCTGCTGCGCTGGCTCAAGGTATGGCTGCACAAGCTACAGTTTCACCTGAGGCTATGGCTGCTGCCGCTGATTCCGTAGGTTTACAGCCGGGAATTTAATACGACTCACTATAGGGAGACCTCATCTTTGAAATGAGCGATGACAAGAGGTTGGAGTCCTCGGTCTTCCTGTAGTTCAACTTTAAGGAGACAATAATAATGGCTGAATCTAATGCAGACGTATATGCATCTTTTGGCGTGAACTCCGCTGTGA‑TGTCtggtg                   <  1:81744/290‑1 (MQ=255)`  
                                                                                                                                                                                                                                                                                       `ggggcaaaCAGTATATGAAAGTGGGTATGGAAAATGGGGATGCTGCGCTGGCTCAAGGTATGGATGAAAAAGCTACAGCTTGACCTGAGGCTATGGCTGCTGCAGCTGAGTCCGTAGGTTTACAGCCGGGAATTTAATACGACTCACTATAGGGAGACCTCATCTTTGAAATGAGCGATGAGAAGAGGTTGGAGTCCTCGGTCTTCCTGTAGTTCAACGTTAAGGAGACAAGAATAAAGGCTGAATCTAATGCAGACGTATATGCATCTTTTGGCGTGAACTCCGCTGTGA‑TGTCtggtgg                  <  1:343495/295‑1 (MQ=255)`  
                                                                                                                                                                                                                                                                                         `gCCCAACAGTCTATGCAAGTGGGTATGGATAATGGTGCTGCTGCGCTGGCTCAAGGTATGGCTGCACAAGCTACAGCTTCACCTGAGGCTATGGCTGCTGCCGCTGATTCCGTAGGTTTACAGCCGGGAATTTAATACGACTCACTATAGGGAGACCTCATCTTTGAAATGAGCGATGACAAGAGGTTGGAGTCCTCGGTCTTCCTGTAGTTCAACTTTAAGGAGACAATAATAATGGCTGAATCTAATGCAGACGTATATGCATCTTTTGGCGTGAACTCCGCTGTGA‑TGTCTGGTGGtt                >  1:100403/1‑301 (MQ=255)`  
                                                                                                                                                                                                                                                                                                 `gTCTATGCAAGTGGGTATGGATAATGGTGCTGCTGCGCTGGCTCAAGGTATGGCTGCACAAGCTACAGCTTCACCTGAGGCTATGTCTGCTGCCGCTGATTCCGTAGGTTTACAGCCGGGAATTTAATACGACTCACTATAGGGAGACCTCATCTTTTAAATTAGCGCTGACAATAGGTTGGAGTCCTCGGTCTTCCTGTAGTTCAACTTTAAGGAGACAATAATAATGGCTGAATCTAATGCAGACGTATATGCATCTTTTGGCGTGCACTCCGCTGTGC‑TGTTTGGTGGTTCCGTTGAg        >  1:163936/1‑301 (MQ=255)`  
                                                                                                                                                                                                                                                                                                 `gTCTATGCAAGTGGGTATGGATAATGGTGCTGCTGCGCTGGCTCAAGGTATGGCTGCACAAGCTACAGCTTCACCTGAGGCTATGGCTGCTGCCGCTGATTCCGTAGGTTTACAGCCGGGAATTTAATACGACTCACTATAGGGAGACCTCATCTTTTAAATGAGCGATGACAATAGGTTGGAGTCCTCGGTCTTCCTGTAGTTCAACTTTAAGGAGACAATAATAATGGCTGAATCTACTGCAGACGTATATGCATCTTTTGGCGTGAACTCCGCTGTGACTGTCtcttatacacatctc         >  1:11103/1‑287 (MQ=255)`  
                                                                                                                                                                                                                                                                                                 `gTCTATGCAAGTGGGTATGGATAATGGTGCTGCTGCGCTGGCTCAAGGTATGGCTGCACAAGCTACAGCTTCACCTGAGGCTATGGCTGCTGCCGCTGATTCCGTAGGTTTACAGCCGGGAATTTAATACGACTCACTATAGGGAGACCTCATCTTTGAAATGAGCGATGACAAGAGGTTGGAGTCCTCGGTCTTCCTGTAGTTCAACTTTAAGGAGACAATAATAATGGCTGAATCTAATGCAGACGTATATGCATCTTTTGGCGTGAACTCCGCTTTGC‑Tgctgtctcttatacacatc        >  1:75782/1‑283 (MQ=255)`  
                                                                                                                                                                                                                                                                                                 `gTCTATGCAAGTGGGTATGGATAATGGTGCTGCTGCGCTGGCTCAAGGTATGGCTGCACAAGCTACAGCTTCACCTGAGGCTATGGCTGCTGCCGCTGATTCCGTAGGTTTACAGCCGGGAATTTAATACGACTCACTATAGGGAGACCTCATCTTTGAAATGAGCGATGACAAGAGGTTGGAGTCCTCGGTCTTCCTGTAGTTCAACTTTAAGGAGACAATAATAATGGCTGAATCTAATGCAGACGTATATGCATCTTTTGGCGTGAACTCCGCTGTGA‑TGTCTGGTGGTTCCGTTGAg        >  1:161547/1‑301 (MQ=255)`  
                                                                                                                                                                                                                                                                                                 `gTCTATGCAAGTGGGTATGGATAATGGTGCTGCTGCGCTGGCTCAAGGTATGGCTGCACAAGCTACAGCTTCACCTGAGGCTATGGCTGCTGCCGCTGATTCCGTAGGTTTACAGCCGGGAATTTAATACGACTCACTATAGGGAGACCTCATCTTTGAAATGAGCGATGACAAGAGGTTGGAGTCCTCGGTCTTCCTGTAGTTCAACTTTAAGGAGACAATAATAATGGCTGAATCTAATGCAGACGTATATGCATCTTTTGGCGTGAACTCCGCTGTGA‑TGTCTGGTGGTTCCGTTGAg        >  1:206031/1‑301 (MQ=255)`  
                                                                                                                                                                                                                                                                                                 `gTCTATGCAAGTGGGTATGGATAATGGTGCTGCTGCGCTGGCTCAAGGTATGGCTGCACAAGCTACAGCTTCACCTGAGGCTATGGCTGCTGCCGCTGATTCCGTAGGTTTACAGCCGGGAATTTAATACGACTCACTATAGGGAGACCTCATCTTTGAAATGAGCGATGACAAGAGGTTGGAGTCCTCGGTCCTCCTGTAGTTCAACTTTAAGGAGACAATAATAATGGCTGAATCTAATGCAGACGTATATGCATCTTTTGGCGTGAACTCCGCTGTGA‑TGTCtggctgtctcttatac        >  1:136884/1‑288 (MQ=255)`  
                                                                                                                                                                                                                                                                                                   `ttATGCAAGTGGGTATGGTTAATGGTGCTGCTGCGCTGGCTTAAGGTATGGCTGCACAAGCTACAGGTTCACCTGAGGCTATGGCTGCTGCCGCTGATTCCGTAGGTTTACAGCCGGGAATTTAATACGACTCACTATAGGGAGACCTCATCTTTGAAATGAGCGATGACAAGAGGTTGGAGTCCTCGGTCTTCCTGTAGTTCAACTTTAAGGAGACAATAATAATGGCTGAATCTAATGCAGACGTATATGCATCTTTTGGCGTGAACTCCGCTGTGA‑TGTCTGGTGGTTCCGTTGAGGa      <  1:305204/300‑1 (MQ=255)`  
                                                                                                                                                                                                                                                                                                       `gCAAGTGGGTATGGATAATGGTGCTGCTGCGCTGGCTCAAGGTATGGCTGCACAAGCTACAGCTTCACCTGAGGCTATGGCTGCTGCCGCTGATTCCGTAGGTTTACAGCCGGGAATTTAATACGACTCACTATAGGGAGACATCATCTTTGAAATGAGCGATGACAAGAGGTTGGAGTCCTCGGTCTTCCTGTAGTTCAACTTTAAGGAGACAATAATAATGGCTGAATCTAATGCAGACGTATATGCATCTTTTGGCGTGAACTCCGCTGTGA‑TGTCTGGTGGTTCCGTTGAGGAACAt  <  1:140818/301‑1 (MQ=255)`  

                                                                                                                                                                                                                                                                                                           `|`                                                                                                                                                                                                                                                                                                           
`CTCTGGTACGAGTGCTCTTGAAGCAACTACAAGCCACGCAACAGATTCCTGAGTTACCTAAGGAAGCCGTAGAGCCAACCATTAGTACAGGTCTGGAAGCAATTGGTCGAGGACAAGACCTTGATAAGCTGGAGCGGTGTGTCACTGCGTGGGCTGCACTGGCACCTATGCGGGACGACCCTGATATTAACCTTGCGATGATTAAGTTACGTATTGCCAACGCTATCGGTATTGACACTTCTGGTATTCTACTCACCGAAGAACAGAAGCAACAGAAGATGGCCCAACAGTCTATGCAAATGGGTATGGATAATGGTGCTGCTGCGCTGGCTCAAGGTATGGCTGCACAAGCTACAGCTTCACCTGAGGCTATGGCTGCTGCCGCTGATTCCGTAGGTTTACAGCCGGGAATTTAATACGACTCACTATAGGGAGACCTCATCTTTGAAATGAGCGATGACAAGAGGTTGGAGTCCTCGGTCTTCCTGTAGTTCAACTTTAAGGAGACAATAATAATGGCTGAATCTAATGCAGACGTATATGCATCTTTTGGCGTGAACTCCGCTGTGA‑TGTCTGGTGGTTCCGTTGAGGAACAT  >  NC_001604.1/21435‑22030`

  

Alignment Legend

Aligned base mismatch/match (shaded by quality score): `ATCG/ATCG` `< 3 ≤` `ATCG/ATCG` `< 11 ≤` `ATCG/ATCG` `< 22 ≤` `ATCG/ATCG` `< 34 ≤` `ATCG/ATCG` `< 38 ≤` `ATCG/ATCG`

Unaligned base: `atcg`    Masked matching base: `atcg`    Alignment gap: `‑`    Deleted base: `‑`
